# Obstructive lingual thyroid

**DOI:** 10.1002/ccr3.3036

**Published:** 2020-08-19

**Authors:** Mario E. Gonzalez

**Affiliations:** ^1^ Department of Otolaryngology Head and Neck Surgery University of Puerto Rico School of Medicine San Juan Puerto Rico

**Keywords:** dysphagia, hypothyroidism, lingual, obstructive, thyroid, tongue

## Abstract

Lingual thyroid should be considered in the differential diagnosis of any base of tongue mass. Examination may reveal worrisome obstructive findings. However, a majority of patients are hypothyroid and respond favorably to hormone replacement therapy upfront. Surgery is reserved for patients unresponsive to medical management or those with severe obstruction.

## Graphical abstract

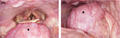

## 1 CASE DISCUSSION

**Figure 1:**
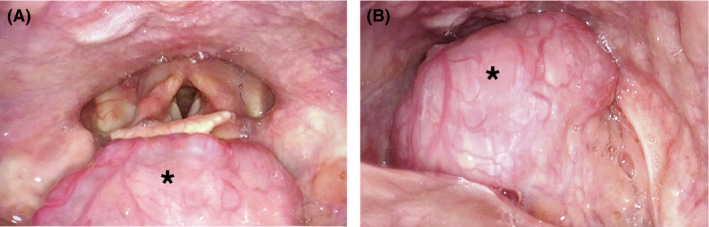
Flexible nasolaryngoscopy showed a round, vascular mass (*) in the base of tongue region. At rest, there was full visualization of the vocal cords (A). Upon initiation of swallowing, posterior displacement of the mass (*) caused airway obstruction above the larynx (B)

A 42‐year‐old woman presented to the clinic with a 3‐month history of dysphagia, orthopnea, and muffled voice. Flexible nasolaryngoscopy showed a round, large vascular mass (*) centered in the base of tongue and vallecula region (Figure 1A). Tongue movement and swallowing caused significant posterior displacement of the mass, with obstruction of the lower pharyngeal airway and the larynx (Figure 1B). Differential diagnosis included malignant neoplasia such as squamous cell carcinoma, adenocarcinoma, or sarcoma, benign or malignant lingual tonsil pathology, and lingual thyroid. Workup was remarkable for hypothyroidism, and CT scan showed a base of tongue homogeneously enhancing mass with absent thyroid tissue in the anterior neck, confirming a diagnosis of lingual thyroid. She is currently symptom‐free, on thyroid hormone replacement therapy, with normalization of thyroid function tests.

This image illustrates the potentially alarming obstructive examination findings of a lingual thyroid. However, up to a third of patients respond to hormone suppression treatment, which should be initially considered in most patients.^1^ Patients with significant symptoms unresponsive to medical therapy or severe symptoms upon presentation should be offered either surgery or radioactive iodine if unfit for general anesthesia.^2^

## CONFLICT OF INTEREST

None declared.

## AUTHOR CONTRIBUTIONS

MG: involved in image and editing, manuscript writing, and review of literature.
